# Declining conventional cancer treatment and using complementary and alternative medicine: a problem or a challenge?

**DOI:** 10.3747/co.v15i0.281

**Published:** 2008-08

**Authors:** M.J. Verhoef, M.S. Rose, M. White, L.G. Balneaves

**Affiliations:** * Department of Community Health Sciences, Faculty of Medicine, University of Calgary, Calgary, AB.; † Calgary Health Region, Calgary, AB.; ‡ Research consultant, Vancouver, BC.; § School of Nursing, University of British Columbia, Vancouver, BC.

**Keywords:** Characteristics of cancer patients, conventional cancer treatment, complementary and alternative medicine, cam, physician responses, control

## Abstract

**Background:**

Several studies have shown that a small but significant percentage of cancer patients decline one or more conventional cancer treatments and use complementary and alternative medicine (cam) instead.

**Objectives:**

Here, drawing on the literature and on our own ongoing research, we describe why cancer patients decide to decline conventional cancer treatments, who those patients are, and the response by physicians to patients who make such decisions.

**Results:**

Poor doctor–patient communication, the emotional impact of the cancer diagnosis, perceived severity of conventional treatment side effects, a high need for decision-making control, and strong beliefs in holistic healing appear to affect the decision by patients to decline some or all conventional cancer treatments. Many patients indicate that they value ongoing follow-up care from their oncologists provided that the oncologists respect their beliefs. Patients declining conventional treatments have a strong sense of internal control and prefer to make the final treatment decisions after considering the opinions of their doctors. Few studies have looked at the response by physicians to patients making such a decision. Where research has been done, it found that a tendency by doctors to dichotomize patient decisions as rational or irrational may interfere with the ability of the doctors to respond with sensitivity and understanding.

**Conclusions:**

Declining conventional treatment is not necessarily an indicator of distrust of the medical system, but rather a reflection of many personal factors. Accepting and respecting such decisions may be instrumental in “keeping the door open.”

## 1. INTRODUCTION

Since the early 1980s, reports have emerged of cancer patients declining conventional cancer treatment and using complementary and alternative medicine (cam) instead (for example, Cassileth *et al.* 1). Although some patients decline all conventional treatments and use cam as an alternative, others decline only some conventional treatments and complement the treatment they accept with cam. For physicians, this choice by a patient is often difficult and troublesome, because it involves risks such as delays in conventional oncology treatments, side effects of cam, and decreased survival time. Many terms have been used to describe this decision—”abandoning,” “non-compliance,” “refusing,” and “rejecting”—most of which carry a negative or pejorative connotation.

How many patients make this decision is not very well known, but the number appears substantial enough to warrant close attention. For example, Cassileth *et al.* 1 examined cancer patients attending a university cancer centre (*n* = 304) and patients recruited through various U.S. media sources who were receiving treatments from cam practitioners or cam clinics (*n* = 356). Of the 378 patients who used cam, 53 (14%) declined conventional treatment of any kind. Another survey 2 revealed that 13% of patients being referred for postsurgical cancer treatment (*n* = 158) rejected all further treatment, and 19% declined some treatments. A third study 3 found that about 3% of women under age 65 with breast cancer (*n* = 302) had refused conventional treatment.

Valid estimates of the prevalence of the choice to decline conventional treatments are not available, but a number of relatively small-scale qualitative studies have focused on understanding why cancer patients make this decision.

In general terms, qualitative research consists of the investigation of phenomena, typically in a detailed and holistic fashion, through the collection of rich narrative materials using a flexible research design 4. Drawing on the literature and on our own ongoing research, we here describe why cancer patients decide to decline conventional cancer treatments, who those patients are, and the response of physicians to patients who make such decisions.

## 2. DISCUSSION

### 2.1 Factors Contributing to the Decision to Decline Conventional Cancer Treatment

In 1999–2000, we conducted a qualitative study with 31 cancer patients who had declined all conventional cancer treatments and were using cam 5. Numerous motivations for this decision were reported, including a negative experience with mainstream medicine, loss of family members or friends to cancer while on conventional treatment, cam use before diagnosis, and a strong belief system in favour of whole-person (holistic) healing. Because these factors existed before the cancer diagnosis, we considered them to be “predisposing.” Factors affecting a decision to decline treatment after diagnosis included poor doctor–patient communication, the emotional effect of the diagnosis, perceived severity of conventional treatment side effects, a high need for decision-making control, and strong beliefs in holistic healing and the mind–body–spirit connection.

In Hawaii, Shumay and colleagues 6 conducted a similar study (*n* = 14) with comparable results. They also identified factors such as beliefs about conventional treatment, the relationship with treatment providers, and beliefs about cam as an alternative treatment option.

In a small, ethnographic study of cancer patients who had declined conventional treatment (*n* = 8), Montbriand described factors similar to those in the previous studies and identified that emotional factors such as anger and fear were commonly expressed during the interviews 7.

Authors van Kleffens and van Leeuwen 8 concluded that medical and personal reasons both play a role in a patient’s decision to refuse treatment, but that personal values and experiences predominate. This study also revealed that patients find quality of life to be very important and seemed to believe that quality of life is incompatible with oncology treatment. They presented a list of 22 reasons why patients refuse recommended conventional cancer treatment, including some including general reasons similar to the ones we found (for example, “want to stay in control”) and more specific motivations, such as not wanting a stoma or loss of a breast, and not wanting to fight any more.

We recently completed a mixed-methods study of 29 men with prostate cancer who declined all conventional treatments 9. A similar study of women with breast cancer declining one or more conventional treatments is currently underway. To date, 32 women have enrolled in this 2-year observational case-controlled study. In-depth baseline interviews were conducted with all participants in both studies. Table I shows the sociodemographic descriptions and treatment choices of the participants.

Findings from the qualitative interviews highlight the extent to which the type of cancer, and possibly sex, influence these choices. In men, we studied the choice to decline all conventional treatments, and in women, the choice to decline at least one conventional treatment. Men and women differed in how they verbalized their treatment decision-making experiences, but both groups raised very similar issues. Foremost, participants described conducting an extensive search for information to evaluate cancer treatment (conventional and cam) options and to make informed choices. Sources of evidence cited by participants included personal experience, scientific evidence (medical literature), anecdotal information, and finding treatment consistent with their health beliefs. Men in particular acknowledged how much their decision was influenced by their perception of the negative experiences shared by other men with prostate cancer who depended on conventional treatments alone. Men and women both cited having control over decision-making and healing approaches as being essential during their cancer experience. They felt that being in control brought on feelings of well-being. Beliefs about conventional medicine (for example, “Western medicine treats the tumour, not the whole person”), cam (for example, “holistic medicine treats the whole person”), and causes of cancer also played a very important role in the decision by men and by women to decline treatment. Although men were found to emphasize the role of spirituality in their treatment decisions and cancer management in more depth than women did, the interplay between mind, body, and spirit was a vital part of the healing approach for men and women alike. Along the same line, physical, emotional, spiritual, and whole-person outcomes of treatment were all considered important indicators of treatment success. Last, in the search for informed treatment decisions, support by family and friends and cam practitioners was highly valued by participants. Cancer specialists were mentioned, but more often support came from family physicians. Men and women both mentioned the huge support received from integrative cancer clinics, which assist patients to make informed choices about the integration of conventional and cam cancer treatments. Many patients also indicated that they valued the ongoing follow-up care from their oncologists provided that they felt supported in their health beliefs. “Keeping the door open” was an important theme that emerged, because most patients wanted to keep their options open. Patients appreciated oncologists who were able to openly communicate that, although they did not agree with the patient’s decision to decline treatment, they would continue to support the patient and provide follow-up care. Conversely, patients who perceived that their cancer specialist was threatening them with a “death sentence,” pressuring them into accepting treatment, or making disparaging comments about cam were more likely to drop out of the conventional cancer system.

Sex differences were also observed in the manner in which participants framed their recommendations for health professionals involved in cancer care. Men mentioned allowing patients sufficient time to adjust to the diagnosis and to make treatment decisions, considering how cancer treatment affects all aspects of well-being; encouraging patients to play an active role in treatment decisions and healing; and being open to assisting patients to find a physician who can support their philosophy of healing. Women identified reducing cancer-related stress at early diagnosis and supporting patients in making the best treatment choices for themselves. They also highlighted that health professionals should pay attention to both the individual woman and the whole person. Men and women both emphasized the need for health professionals to be aware of and to refer patients to integrative cancer care clinics or services.

### 2.2 Psychosocial Characteristics of Patients Declining Some or All Conventional Treatments

In the prostate and breast cancer studies, we used the Multiple Health Locus of Control (mhlc) scale 10, the General Self-Efficacy (gse) scale 11, and the Control Preferences Scale (cps) 12 to assess psychosocial characteristics of participants. The mhlc scale measures the degree to which people believe that internal resources or external factors such as luck, chance, doctors, or powerful others affect their disease outcome. The gse scale assesses an individual’s perceived sense of general self-efficacy and is suitable for studies examining adaptation after a life change or a stressful event. The cps allows for the identification of the role (that is, active, collaborative, passive) that patients wish to play in disease management and treatment decisions.

Table II presents the results from the mhlc scale, in which the means and 95% confidence intervals (cis) for the four subscales completed by the prostate cancer and breast cancer patients who declined conventional treatment are compared to published normative data from patients with a diagnosis of cancer 10. Men and women who declined conventional cancer treatment both had scores on the internal scale that were higher than the published normative data from patients with a diagnosis of cancer, but scores on the chance, doctors, and powerful others scales that were lower.

Scores on the gse scale were higher both for the prostate cancer group (mean: 34.8; 95% ci: 33.4 to 36.2) and for the breast cancer group (mean: 32.3; 95% ci: 30.9 to 33.7) than the normative scores for the American adult population (mean: 29.4; 95% ci: 9.2 to 29.7) 11.

The two study groups were similar on the cps, with none of the participants in either group preferring to play a passive role and have the doctor make the final decision (Fisher exact test: *p* = 0.100). Men and women were both most likely to prefer an active role and to make the final decision after seriously considering the opinions of their doctors (72% for the breast cancer group, 46% for the prostate cancer group). Only 16% of the breast cancer group and 21% of the prostate cancer group indicated that they would prefer to make the final decision about their treatment. With regard to collaborative decision-making, 32% of the prostate cancer group indicated that they preferred decision-making to be shared between them and their doctors, but only 12% of the breast cancer group preferred that option.

### 2.3 Response of Physicians to Patients Who Decline Conventional Cancer Treatment

Little is known about how physicians regard the choices patients make, in particular when patients decide to decline potentially curative treatments. Again, work in this area is mostly qualitative. Authors van Kleffens and van Leeuwen 8 assessed how oncologists and general practitioners (*n* = 16) evaluated such a decision by a patient. They found that although patients base their decisions mostly on personal values or experience, physicians emphasize a goal-oriented medical perspective. From the point of view of the doctors, the decision to decline conventional treatment appears irrational, especially when the proposed treatment is curative. In the case of palliative treatment, physicians have less difficulty accepting the patient’s decision.

Recently, Madjar *et al.* 13 followed up on this notion in a qualitative study of medical and radiation oncologists (*n* = 12) and found that physicians tend to view (“construct”) patients and their decisions in terms of mutually exclusive categories. In addition to distinguishing between curable and non-curable diseases, and between rational and irrational treatment decisions, physicians also distinguished between patients who took a passive or an active role in decisions. Although most patients will go along with their physician’s recommendation and are fairly passive in decision-making, active patients are perceived to be different and possibly to seek alternative health options for which limited scientific evidence is available, and sometimes to decline conventional treatments. It is thus not surprising that some physicians see patients who decline conventional treatment in favour of cam as difficult, irrational patients who require extra time and challenge physicians’ authority. What physicians consider to be the salient features of the situation, such as the nature of the disease, the nature of the patient’s decision, and the personal characteristics of the patient, is in each of these studies characterized by a dichotomy.

It is important to acknowledge the feelings, concerns, and reflections of physicians about their role when faced with patients who wish to take an active role in decision-making and to pursue alternative options to conventional care. The main themes arising from interviews with physicians were feelings of uncertainty, of failure (for example, failure to understand or to get to the bottom of the problem), of helplessness, and of concern (about the patient and the implications of the patient’s decision). According to Madjar *et al.,* the tendency of the physicians to perceive a patient’s decision to decline conventional treatment as either a rational or irrational decision may contribute to such feelings of uncertainty and concern, and may interfere with the ability of physicians to respond to such decisions with sensitivity and understanding.

## 3. CONCLUSIONS

The picture that emerges from studying people who decline conventional treatments is not necessarily one of “problem patients,” but of a unique group of self-directed, confident, and active patients who have thought deeply about the meaning of cancer and about their cancer treatment options. It may not always be easy for clinicians to deal with these patients as they deviate from the norm and challenge current evidence, but in the end, relationships with these patients can be rewarding and insightful.

Without exception, we found that these patients spend much time researching their treatment options. The sources of information they use reflect, to some degree, the definition of evidence set out by Sackett *et al.* 14, which emphasizes the integration of best available evidence from systematic research, professional judgment, and patient values. In this context, patients ideally make treatment decisions that are informed by evidence; that meet their values, beliefs, and expectations; and that are supported by the clinical expertise of (conventional and cam) practitioners. For many patients, individual authority and the “lived experience” are also emerging as valuable information sources.

The rationality of the decision by patients to decline some or all conventional cancer treatments has been discussed in the literature. For example, Huijer and van Leeuwen 15 concluded that what might appear to be an irrational decision in a medical context actually results from a balancing process in the patient’s personal context over time. This point has also been discussed by Kingston 16, who indicates that these patients are often erroneously labelled “difficult”: “Horses refuse at a jump, badly behaved dogs refuse to obey their masters. Our patients, I hope, make decisions.”

We have also identified a desire on the part of patients to be in control of the treatment decision-making process and a belief in their own ability to successfully exercise influence over events that affect their lives (“self-efficacy”). Clearly, both concepts are closely related. These findings are consistent with literature focused on personality characteristics of patients who use cam. The most common themes in these studies suggest that cam users are more open (creative, imaginative, intelligent) than the general population 17,18 and that they desire a more active role in decision-making 18–20.

Despite the important recommendations that patients have provided regarding the role of health professionals in decisions related to conventional care and cam, other factors need to be considered as well. Treatment decision-making by patients is a process not limited to one point in time; it will depend on many different factors. In the prostate cancer study, we found that, within the 3-year follow-up period, 5 of the men eventually decided to use some form of conventional treatment.

The need for effective, compassionate, open-minded, and respectful communication is probably the most important theme in the studies we have reviewed (for example, Shumay *et al.* 6, Montbriand 7). Alleviating patient concerns about conventional cancer treatments, understanding the potential supportive role of cam, being aware of patient preferences, and the personality characteristics of patients related to decision-making is crucial. In several studies, poor communication was even mentioned as a reason for declining conventional treatment (for example, Shumay *et al.* 6). Understanding who these patients are and what their motivations are may help to improve communication. In addition, it is important to keep in mind that most patients want to discuss these issues with their physicians and prefer to stay in touch. The decision to decline treatment is not necessarily an indicator of distrust of the medical system and the care received to date, but can be a reflection of intensely personal factors. Accepting the challenge and recognizing and honouring the uniqueness of patients who decline conventional treatments will create opportunities for rich patient–provider relationships that will transform “problem” patients into partners in care.

**TABLE I:** Sociodemographic characteristics of prostate (*n* = 29) and breast (*n* = 33) cancer patients

*Characteristic*	*Patients (*n*) with cancer of*	
	*prostate*	*breast*
Age		
40–49	0	11
50–59	7	15
60–69	8	5
≥70	14	2
Marital status
Married	23	
Married or living with a partner		19
Other	6	14
Education level		
High school or less	6	10
Technical or some university	11	7
University degree or higher	12	16
Employment status		
Employed or self-employed	14	18
Retired	14	6
Unemployed	1	7
Top 3 conventional treatments declined
Surgery	21	
Radiation therapy	12	22
Brachytherapy	9	
Chemotherapy		22
Hormone therapy		20

**TABLE II:** Scores on the Multiple Health Locus of Control scale for prostate and breast cancer patients compared with normative scores for patients with a diagnosis of cancer

*Item*	*Group*	n	*Mean*	*SD*	*95% CI*
Internal	Cancer diagnostic group a	93	18.5	5.72	17.3 to 19.7
	Prostate cancer group	29	27.1	5.46	25.1 to 29.2
	Breast cancer group	33	22.7	6.55	20.4 to 25.0
Chance	Cancer diagnostic group a	93	19.8	7.13	18.3 to 21.3
	Prostate cancer group	29	14.9	6.58	12.4 to 17.4
	Breast cancer group	33	12.6	5.42	10.7 to 14.6
Doctors	Cancer diagnostic group a	93	15.9	2.39	15.4 to 16.4
	Prostate cancer group	29	10.7	3.42	9.4 to 12.0
	Breast cancer group	33	8.9	3.85	7.5 to 10.2
Powerful others	Cancer diagnostic group a	93	11.0	3.96	10.1 to 11.8
	Prostate cancer group	29	8.1	2.55	7.1 to 9.1
	Breast cancer group	33	5.9	2.69	5.0 to 6.9

^a^ Wallston *et al.* 10.

SD = standard deviation; CI = confidence interval.
